# Colonization of multidrug-resistant Gram-negative bacteria increases risk of surgical site infection after hemorrhoidectomy: a cross-sectional study of two centers in southern China

**DOI:** 10.1007/s00384-023-04535-1

**Published:** 2023-10-02

**Authors:** Jian-guo Li, Li-lian Gao, Cun-chuan Wang, Jia-min Tu, Wen-hui Chen, Xiang-lin Wu, Jin-xia Wu

**Affiliations:** 1 Department of Colorectal Surgery, First Affiliated Hospital of Jinan University, Guangzhou, 510630 China; 2 Department of Colorectal Surgery, University of Chinese Academy of Sciences Shenzhen Hospital, Shenzhen, China

**Keywords:** Colorectal surgery, Hemorrhoidectomy, Multidrug-resistant Gram-negative bacteria, Surgical site infection, Rectal swab

## Abstract

**Purpose:**

The present study aims to determine the rectoanal colonization rate and risk factors for the colonization of present multidrug-resistant bacteria (MDRBs). In addition, the relationship between MDRB colonization and surgical site infection (SSI) following hemorrhoidectomy was explored.

**Methods:**

A cross-sectional study was conducted in the Department of Colorectal Surgery of two hospitals. Patients with hemorrhoid disease, who underwent hemorrhoidectomy, were included. The pre-surgical screening of multidrug-resistant Gram-negative bacteria (MDR-GNB) colonization was performed using rectal swabs on the day of admission. Then, the MDRB colonization rate was determined through the rectal swab. Logistic regression models were established to determine the risk factors for MDRB colonization and SSI after hemorrhoidectomy. A *p*-value of < 0.05 was considered statistically significant.

**Results:**

A total of 432 patients met the inclusion criteria, and the MDRB colonization prevalence was 21.06% (91/432). The independent risk factors for MDRB colonization were as follows: patients who received ≥ 2 categories of antibiotic treatment within 3 months (odds ratio (OR): 3.714, 95% confidence interval (CI): 1.436–9.605, *p* = 0.007), patients with inflammatory bowel disease (IBD; OR: 6.746, 95% CI: 2.361–19.608, *p* < 0.001), and patients with high serum uric acid (OR: 1.006, 95% CI: 1.001–1.010, *p* = 0.017). Furthermore, 41.57% (37/89) of MDRB carriers and 1.81% (6/332) of non-carriers developed SSIs, with a total incidence of 10.21% (43/421). Based on the multivariable model, the rectoanal colonization of MDRBs (OR: 32.087, 95% CI: 12.052–85.424, *p* < 0.001) and hemoglobin < 100 g/L (OR: 4.130, 95% CI: 1.556–10.960, *p* = 0.004) were independently associated with SSI after hemorrhoidectomy.

**Conclusion:**

The rectoanal colonization rate of MDRBs in hemorrhoid patients is high, and this was identified as an independent risk factor for SSI after hemorrhoidectomy.

## Introduction

Hemorrhoidal disease is one of the most prevalent proctological diseases, making hemorrhoidectomy one of the most common surgical procedures performed in colorectal surgery. Antibiotic prophylaxis is uniformly recommended for all clean-contaminated, contaminated, and dirty procedures, including hemorrhoidectomy [1]. However, the widely used antibiotic prophylaxis may result in antibiotic resistance.

A decade ago, postoperative surgical site infection (SSI) was considered an exceedingly rare event following hemorrhoidectomy [2]. However, in recent years, there has been a significant increase in the proportion of SSIs following colorectal surgery, and the efficacy of antibiotic prophylaxis agents in preventing SSI following colorectal surgery has declined in recent years [3, 4]. The reduction in efficacy can be explained by the increase in intestinal colonization of multidrug-resistant Gram-negative bacteria (MDR-GNB) [5]. As a result, medical burden has significantly increased due to MDRB colonization, and this has been recognized as a matter of particular concern in colorectal surgery.

In 2017, the World Health Organization (WHO) published a list of bacteria that urgently need new antibiotics, and the most critical group was MDR-GNB, which poses a particular threat. Carbapenem-resistant *Acinetobacter baumannii* (CRAB), carbapenem-resistant *Pseudomonas aeruginosa* (CRPA), carbapenem-resistant Enterobacterales (CRE), such as carbapenem-resistant *Klebsiella pneumoniae* (CRKP), and extended-spectrum β-lactamase-producing Enterobacterales (ESBL-PE) are the main MDR-GNBs in the world [6]. Fecal carriage of ESBL-PE increases the infection risk after liver transplant [7]. Compared with non-carriers, ESBL-PE carriers have significantly higher SSIs after colorectal surgery (24.8% vs. 11.1%, *p* < 0.001) [8]. Furthermore, a systematic review and meta-regression analysis revealed that the pooled cumulative incidence of infection was 14% at a median follow-up time of 30 days for MDR-GNB, while the infection risk was 19% for patients colonized with CRE and 8% for patients with ESBLS-PE [9]. Patients colonized with MDRBs are the bacteria reservoirs in hospitals, which can potentially ignite the explosion of fatal infection outbreaks. Thus, the active screening of MDRB colonization through a simple method has important value [10–12]. Rectal swabs can be easily and immediately obtained and stored in a standardized fashion, without previous perturbation of the microbiota. Furthermore, rectal swabs are already widely used for screening resistant microbes, and these have been shown to be very effective [13, 14].

MDRB gastrointestinal colonization is not uncommon worldwide, at present [15–18]. Furthermore, the detection rate of MDRBs has significantly increased in China [19]. Numerous studies have demonstrated that MDRB colonization and healthcare-associated infections (HAIs) are associated with subsequent infections [20–22], but the factor for determining this progression remains unclear. Hemorrhoidectomy is a common surgical method, but the impact of the rectoanal colonization of MDRBs in patients who underwent hemorrhoidectomy remains unclear.

The present study generally aims to determine the rectoanal colonization rate of present MDRBs and its risk factors and explore the relationship between MDRB colonization and SSI after hemorrhoidectomy.

## Methods

### Study design, patients, and setting

A two-center cross-sectional study was conducted. Patients with hemorrhoidal disease, who were hospitalized in the Department of Colorectal Surgery from June to August 2022, were enrolled. Patients with existing infection in the rectoanal area at the time of admission were excluded. All patients underwent a Milligan-Morgan open hemorrhoidectomy with a harmonic scalpel. During the study period, environmental monitoring was conducted once a month in the ward. Nine surfaces (bedrails, privacy curtains, carts, bedside table, commodes, doorknobs, fumigation basins, faucet handles, and shared medical equipment) were surveyed monthly in two rooms after terminal cleaning. MDRB colonization or infection patients received standard prophylaxis and contact isolation after being diagnosed. The study protocol applied for the two hospitals was approved by the ethics committee of these hospitals.

### Data collection

The demographic and clinical data were initially collected using the electronically digitized medical records of patients. The collected patient information included the following: age, gender, hemorrhoid diagnosis (internal, external, or mixed), and comorbid medical conditions (hemoglobin (Hb) < 100 g/L, diabetes, hypertension, hyperuricemia, inactive inflammatory bowel disease (IBD), smoking history, current steroid use, medical events in the past 3 months (hospitalization for ≥ 7 days and ≥ 2 categories of antibiotic treatment), prophylactic antibiotic regimens, the presence or absence of SSI, and the infection site (superficial, deep, or organ/space)).

A bacterial resistance surveillance system (Xinglin Technology) was used to monitor the MDRBs. The surveillance data (multidrug-resistant strains and susceptibility test results) was automatically fed back to the clinical physicians in real time via the intranet and to the China Antimicrobial Resistance Surveillance System (CARSS). If any data was missing, these were obtained through outpatient clinic reviews or telephone conversations within 1 month.

### Definitions

The combination of the Centers for Disease Control and Prevention (CDC) case definitions and clinical judgment were used to differentiate between infection and colonization [23]. The criterion was superficial incisional, deep incisional, or organ-space infection [24]. Patients with fever, persistent or worsening pain, and signs of local inflammation, drainage, and/or wound splitting were defined as having superficial SSI, while patients who required incision and drainage were defined as having deep SSI. The hemorrhoid diagnosis was categorized as internal (proximal to the dentate line), external (distal to the dentate line), or mixed (both proximal and distal) [25]. Inactive IBD means that the disease is steroid-free and in clinical and biochemical remission. For the prophylactic antibiotic, cefuroxime, cefazolin, clindamycin, or metronidazole was used to cover the major pathogenic bacteria. MDRB was defined as a bacteria that is resistant to at least one agent in three or more antimicrobial categories [26]. According to the list of antibiotic-resistant priority pathogens published by the WHO in 2017, the following critical MDRBs were surveyed: (i) ESBL-PE, (ii) CRE, (iii) CRAB, and (iv) CRPA. Patients with fecal carriage of MDRBs were referred to as RS(+), while patients who did not have this were referred to as RS(−).

### Microbiological studies

Rectal swabs were performed by a trained nurse on the day of admission (within 24 h). Microbiological samples were taken from the wounds and/or peritoneal fluid or abscesses for culture in patients with suspected SSI prior to the use of therapeutic antibiotics. The matrix-assisted laser desorption/ionization-time of flight (MALDI-TOF)-mass spectrometry (MS) technique was used for pathogen identification. Then, screening tests for extended-spectrum β-lactamases (ESBL) and carbapenemase production were performed by broth microdilution. Afterwards, the synergy test (ceftazidime, cefotaxime, ceftazidime-clavulanate, and cefotaxime-clavulanate) was used as the confirmatory test for ESBL producers, while imipenem, ertapenem, or meropenem was used to screen for carbapenemase production. Subsequently, the modified carbapenem inactivation method (mCIM) assay was used to detect enzymes that degrade carbapenemases. *Escherichia coli* (ATCC 25922), *Enterococcus* (ATCC29212), and *Klebsiella pneumoniae* (ATCC 700603) were used as the reference strains. The screening tests and interpretation of results were conducted according to the Clinical and Laboratory Standards Institute (CLSI) [27]. The drug susceptibility test was routinely performed through the broth microdilution method using the Sensititre™ GNX2F (Thermo Fisher Scientific, Waltham, MA, USA), according to the European Committee on Antimicrobial Susceptibility Testing (EUCAST) criteria [28].

The main outcome measures for the present study were the rectoanal MDRB colonization rate and its risk factors and the overall SSI rate (superficial, deep incisional, and organ/space infection rates). The secondary outcome measures included the microbiology of colonization and infections and the antimicrobial susceptibility testing outcomes.

### Statistical analysis

All statistical analyses were carried out using SPSS 26.0 (IBM, NY, USA). Continuous variables were analyzed using *t*-test or nonparametric tests, and categorical variables were analyzed using chi-square test or Fisher’s exact probabilities. The factors associated with MDRB colonization and SSI were analyzed by binary logistic regression. These were expressed in odds ratios (ORs) with the corresponding 95% confidence intervals (CIs). A *p*-value of < 0.05 was considered statistically significant.

## Results

### Demographic characteristics

A total of 468 patients with hemorrhoids were admitted during the study period. Among these patients, 36 patients had rectoanal inflammation or anal fistula at admission. Thus, a total of 432 patients were enrolled for the present study. The mean age of these patients was 37.63 ± 9.31 years old, and 66.90% (289/432) of these patients were male. Furthermore, 21.06% (91/432) of these patients were identified with MDRB colonization (two patients with multiple MDRBs). An overview of the demographic and perioperative information of the cohort of patients is presented in Table 1.

**Table 1:** Demographic data of all patients (*n* = 432)

**Variable**	**Total,** ***n*** **= 432 (%)**	**RS(+), *n* = 91 (%)**	**RS(−),** ***n* = 341 (%)**	***t/z/x***^***2***^	***p***
Gender					
Male	289 (66.9)	58 (63.7)	231 (67.7)	0.520	0.471
Female	143 (33.1)	33 (36.3)	110 (32.3)		
Age (years, mean ± SD)		39.510 ± 8.786	37.130 ± 9.395	2.167	0.031
< 25	23 (5.3)	2 (2.2)	21 (6.2)	6.045	0.118
25–34	151 (34.9)	28 (30.8)	123 (36.1)		
35–44	163 (37.7)	34 (37.4)	129 (37.8)		
≥ 45	95 (22.0)	27 (29.7)	68 (19.9)		
Current steroid use	8 (1.9)	3 (3.3)	5 (1.5)	0.509	0.478
Comorbidities					
Hb < 100 g/L	61 (14.1)	9 (9.9)	52 (15.2)	1.388	0.239
Diabetes	34 (7.9)	8 (8.8)	26 (7.6)	0.135	0.713
Hypertension	33 (7.6)	9 (9.9)	24 (7.0)	0.828	0.363
IBD	18 (4.2)	10 (11.0)	8 (2.3)	13.438	< 0.001
ME (within 3 months)					
Hospitalization	18 (4.2)	5 (5.5)	13 (3.8)	0.509	0.476
AT (≥ 2 categories)	24 (5.6)	10 (11.0)	14 (4.1)	6.487	0.011
SUA (µmol/L, mean ± SD)		378.21 ± 103.11	330.99 ± 95.62	4.116	< 0.001
Hyperuricemia	124 (28.7)	41 (45.1)	83 (24.3)	15.061	< 0.001
History of smoking	95 (22.0)	21 (23.1)	74 (21.7)	0.079	0.778
Open hemorrhoidectomy	421 (97.5)	89 (97.8)	332 (97.4)	-	-

*SD* standard deviation, *Hb* hemoglobin, *IBD* inflammatory bowel disease, *ME* medical events, *AT* antibiotic treatment, *SUA* serum uric acid

### Multidrug-resistant bacteria colonization rate and risk factor analysis

A total of 93 colonized MDRB strains were identified. ESBL-*Escherichia coli* (ESBL-EC; 72, 77.42%) was the main colonized MDRB, followed by ESBL-*Klebsiella pneumoniae* (ESBL-KP; 15, 16.13%) and CRE (six, 6.45%). In the multivariable regression analysis, ≥ 2 categories of antibiotic treatment within 3 months (OR: 3.714, 95% CI: 1.436–9.605, *p* = 0.007), IBD (OR: 6.746, 95% CI: 2.321–19.608, *p* < 0.001), and high serum uric acid (OR: 1.006, 95% CI: 1.001–1.010, *p* = 0.017) were the major risk factors for the MDRB colonization (Table 2).

**Table 2:** Multivariable regression analysis of risk factors for RS(+)

**Variable**	**OR**	**95% Cl**	***p***
Gender (male)	0.858	0.497–1.480	0.581
Age	1.023	0.997–1.050	0.086
History of smoking	1.561	0.834–2.922	0.164
Comorbidities			
Hb < 100 g/L	0.568	0.251–1.285	0.174
Diabetes	1.320	0.541–3.222	0.542
IBD	6.746	2.321–19.608	< 0.001
ME (in the past 3 months)			
Hospitalization	1.267	0.414–3.879	0.678
AT (> 2 categories)	3.714	1.436–9.605	0.007
SUA	1.006	1.001–1.010	0.017
Hyperuricemia	1.307	0.518–3.300	0.571
Current steroid use	0.277	0.059–1.290	0.102

*OR* odds ratio, *95% CI* 95% confidence interval, *Hb* hemoglobin, *IBD* inflammatory bowel disease, *ME* medical events, *AT* antibiotic treatment, *SUA* serum uric acid

### The prevalence of surgical site infection

Among the 432 patients, 11 patients were treated conservatively or with rubber band ligation, while 421 patients underwent open hemorrhoidectomy. The antibiotic prophylaxis utilization was 44.65% (188/421), and 43 patients (41 superficial incisional and two deep incisional) had documented SSIs. The overall incidence of SSI was 10.21%. The two deep incisions diagnosed in RS(+) patients were treated with incision drainage and anti-infective therapy. There was no significant difference in the prevalence of SSIs between patients who received and did not receive prophylactic antibiotics (9.1% vs. 11.2%, *X*^2^ = 0.483, *p* = 0.487).

For patients who underwent open hemorrhoidectomy, SSI occurred in 41.57% (37/89) of MDRB carriers and 1.81% (6/332) of non-carriers (41.57% vs. 1.81%, *X*^2^ = 121.023, *p* < 0.001). Furthermore, two of the six non-carrier patients subsequently developed MDRB infection (ESBL-EC SSI). RS(+) (OR: 32.087, 95% CI: 1.556–10.960, *p* < 0.001) and Hb < 100 g/L (OR: 4.130, 95% CI: 12.052–85.424, *p* = 0.004) were independently associated with SSI. The univariate and multivariate analysis for SSI is presented in Table 3.

**Table 3:** Univariate and multivariate analysis of risk factors for postoperative infection in patients who underwent open hemorrhoidectomy (*n* = 421)

**Variable**	**Presence of SSI (*n* = 43)**	**Absence of SSI (*n* = 378)**	**Univariate analysis,** ***p***	**Multivariate analysis**		
				**OR**	**95% Cl**	***p***
Gender (male)	25 (58.14%)	256 (67.72%)	0.206			
Age (years; mean ± SD)	37.98 ± 8.32	37.64 ± 9.35	0.822			
History of smoking	9 (20.93%)	85 (22.49%)	0.816			
Comorbidities						
Hb < 100 g/L	13 (30.23%)	46 (12.17%)	0.001	4.130	1.556–10.960	0.004
Diabetes	6 (13.95%)	24 (6.35%)	0.128			
Hypertension	4 (9.30%)	27 (7.14%)	0.837			
IBD	9 (20.93%)	8 (21.16%)	< 0.001	3.070	0.901–10.457	0.073
Hemorrhoid diagnosis						
Internal	13 (30.23%)	153 (40.48%)				
External	6 (13.95%)	52 (13.76%)				
Mixed	24 (55.81%)	173 (45.77%)	0.286			
SUA (µmol/L, mean ± SD)	385.60 ± 109.98	337.83 ± 96.93	0.003	0.998	0.991–1.004	0.504
Hyperuricemia	23 (53.49%)	101 (26.72%)	< 0.001	2.866	0.718–11.443	0.136
Current steroid use	2 (4.65%)	5 (1.32%)	0.154			
Diarrhea	8 (18.60%)	25 (6.61%)	0.013	1.604	0.510–5.043	0.419
Prophylactic antibiotics	17 (39.53%)	171 (45.24%)	0.476			
RS(+)	37 (86.05%)	52 (13.76%)	< 0.001	32.087	12.052–85.424	< 0.001

*SSI* surgical site infection, *OR* odds ratio, *95% CI* 95% confidence interval, *SD* standard deviation, *Hb* hemoglobin, *IBD* inflammatory bowel disease, *SUA* serum uric acid

### Drug sensitivity results

**Fig. 1:**
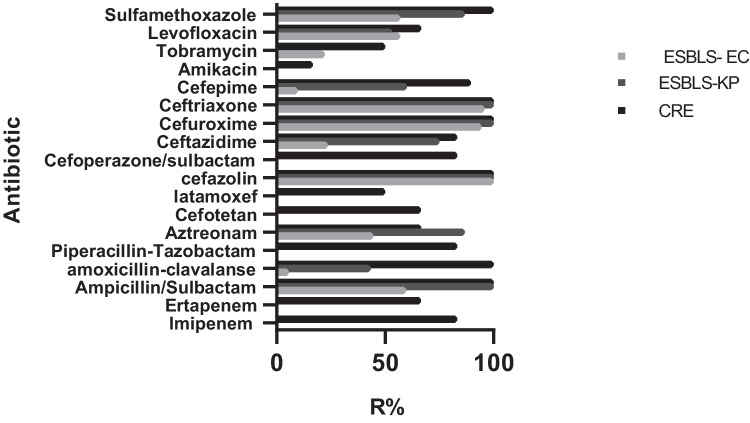
Results for the antimicrobial susceptibility test (resistance rate). ESBL-EC, extended-spectrum β-lactamase-producing *Escherichia coli*; ESBL-KP, extended-spectrum β-lactamase-producing *Klebsiella pneumoniae*; CRE, carbapenem-resistant Enterobacteriaceae

For ESBL-EC, drugs with a resistance rate of > 90% were as follows: cefazolin (100%), cefuroxime (94.44%), and ceftriaxone (95.83%). Drugs with a sensitivity rate of > 90% were as follows: meropenem (100%), imipenem (100%), piperacillin-tazobactam (100%), amikacin (100%), latamoxef (93.06%), and cefepime (90.28%). For ESBL-KP, drugs with a resistance rate of > 90% were as follows: ampicillin/sulbactam (100%), cefazolin (100%), cefuroxime (100%), and ceftriaxone (100%). Drugs with a sensitivity rate of > 90% were as follows: meropenem (100%), imipenem (100%), piperacillin-tazobactam (100%), amikacin (100%), and cefotetan (100%). For CRE, drugs with a resistance rate not higher than 50% were as follows: latamoxef (50.00%), amikacin (16.67%), and tobramycin (50.00%). These above results are presented in Fig. 1.

### Monthly environmental monitoring results

In June 2022, the ESBL-EC strains were observed to colonize on privacy curtains and fumigation basins in the wards. In July 2022, ESBL-EC strains were observed to be colonized on fumigation basins in the wards.

## Discussion

The present study suggests that active screening and early identification of patients with MDRB colonization is of great significance for the prevention of postoperative incision infection and the outbreak of MDRB in the ward. Furthermore, the present study provides epidemiological information for the surgical treatment of colorectal cancer and presents the rational use of antibacterial drugs during colorectal surgery. In the present study, the rectoanal MDRB colonization rate was 21.06%, and the main MDRB was ESBL-PE. The prevalence of MDRB colonization in the present study cohort was higher, when compared to the prevalence in another prospective cohort study conducted within 2012–2017 [8]. However, the present results are consistent with the research results reported by studies conducted within the past 2 years [29, 30]. The following were determined to be risk factors for intestinal MDRB colonization: ≥ 2 categories of antibiotic treatment within 3 months, IBD, and serum uric acid. The close relationship among antibiotics, dysregulated intestinal immunity, intestinal MDRB colonization, and hyperuricemia has been well documented in other studies [31, 32].

In the present study, the MDRB colonization led to the development of MDRB SSI in a significant number of patients, and this became the major cause of postoperative infection after hemorrhoidectomy. That is, SSI occurred in 41.57% of MDRB carriers and 1.81% of non-carriers. In a 2018 study, 38% of SSIs after colorectal surgery were determined to be caused by ESBL-PE [33]. Due to the short hospital stay in our hospital, most of the SSI patients were identified through outpatient reviews and follow-ups. Thus, the SSI rate after hemorrhoid surgery may have been underestimated.

SSIs after hemorrhoidectomy are mostly focal infections, which are marked by more intense wound pain and longer wound healing times. In an extremely limited number of patients, deep infections that are not treated promptly can be fatal [34, 35]. In clinical studies, MDRB infections have been associated with prolonged wound non-healing [36]. Thus, choosing the right antibiotic is the key for the successful treatment of SSIs. According to the drug sensitivity results in the present study, carbapenems should be selected when treating ESBL-PE bloodstream infections. For ESBL-PE focal infections, optional enzyme inhibitor combinations, such as piperacillin/tazobactam, cefotetan, amikacin, and cefoperazone/sulbactam, should be used with caution, while penicillin and cephalosporin without enzyme inhibitors are contraindicated. These results may provide evidence for empirical drug use.

In the meta-analysis conducted by Karanika et al., the prevalence of fecal colonization with ESBL-PE in healthy individuals varied from 2 to 46% by geographic region, with a 5.4% annual increase in prevalence [37]. The colonization of MDRB leads to a high probability of nosocomial MDRB infection, especially in the intensive care unit (ICU) [38, 39]. Hemorrhoidectomy is a kind of contamination operation, and MDRB colonization may lead to MDRB infection, as identified in the present study. In order to prevent MDRB infection, prophylactic antibiotics may be effective. Prophylactic antibiotics previously had no role in cases of hemorrhoidectomy, because SSI following hemorrhoidectomy was a relatively rare event decades ago [2, 40]. Due to the increase in colonization rate of MDRBs, the risk of SSI following colorectal surgery in MDRB carriers who received cephalosporin-based prophylaxis (which does not cover MDRBs) has increased [8]. The screening for colonization offers a potential window of opportunity for the intervention and prevention of SSIs. The early standard prophylaxis and contact isolation of MDRB carriers can effectively prevent the spread of infection from one patient to another, allowing for infection control [41]. Studies have revealed that high-risk patients for MDRB colonization may benefit from preoperative screening [12, 42] and that rectal swab is a remarkable method for active surveillance [43, 44]. However, it remains unclear how MDRB colonization can be managed. The decolonization of MDRBs may reduce subsequent infections. However, this is not routinely recommended due to the lack of long-term efficacy and potential risk of antibiotic resistance [45].

HAIs were common in clinical practice. A meta-analysis study revealed that the prevalence of HAIs in China was 3.12% [46]. In the present study, two RS(−) patients developed ESBL-EC superficial incisional SSIs, and these were determined to be nosocomial infections. Furthermore, the monthly environmental monitoring results revealed that MDRBs colonized in privacy curtains and fumigation basins in the wards. The source of infection for the two RS(−) patients may be associated to environmental or medical intervention. Thus, the risk of infection from environmental and medical interventions should not be ignored.

MDRB colonization and conspicuous subsequent infection would continue to be a daunting challenge in the field of colorectal surgery [47]. The various factors that influence the occurrence and transmission of MDRBs include the following: the use of antimicrobial agents, the level of disinfection and isolation, hand hygiene compliance, and environmental hygiene. A screening system that combines all these factors may be beneficial in the management of MDRBs. An antimicrobial stewardship (AMS) workflow was recommended in a retrospective study [48]. The investigators in that study reported that the workflow led to a significant improvement in appropriate therapy for multidrug-resistant *Pseudomonas* and CRE infections.

The main limitation of the present study was the relatively limited number of participants. However, due to the cross-sectional design of the study, statistically significant results were attained even with the limited number of participants. The technical limitation of the study was that polymerase chain reaction (PCR) and DNA sequencing were not performed to detect the antimicrobial resistance genes, since its characterization is essential for surveillance, infection control, and therapeutic purposes. In future studies, genome-wide sequencing would be performed to identify pathogens, compare the sequence to a database of known pathogens, and identify the closest relatives.

## Conclusion

The incidence of SSI following colorectal surgery has significantly increased in recent years with the emergence of MDRB colonization. The present antibiotic regimen (cephalosporins plus metronidazole) does not cover MDRBs. Monitoring patients with high risk of MDRB colonization, taking measures for isolation, the timely detection and identification of MDRB infection, and sensitive antibiotic treatment are recommended as essential measures.

## Data Availability

The datasets used and/or analyzed in the study are available from the corresponding author upon reasonable request.

## References

[1] Woods RK, Dellinger EP (1998). Current guidelines for antibiotic prophylaxis of surgical wounds. Am Fam Physician.

[2] Nelson DW, Champagne BJ, Rivadeneira DE (2014). Prophylactic antibiotics for hemorrhoidectomy: are they really needed?. Dis Colon Rectum.

[3] Smith R, Coast J (2013). The true cost of antimicrobial resistance. BMJ.

[4] Teillant A, Gandra S, Barter D, Morgan DJ, Laxminarayan R (2015). Potential burden of antibiotic resistance on surgery and cancer chemotherapy antibiotic prophylaxis in the USA: a literature review and modelling study. Lancet Infect Dis.

[5] Kirby A, Santoni N (2015). Antibiotic resistance in Enterobacteriaceae: what impact on the efficacy of antibiotic prophylaxis in colorectal surgery?. J Hosp Infect.

[6] Tacconelli E, Carrara E, Savoldi A (2018). Discovery, research, and development of new antibiotics: the WHO priority list of antibiotic-resistant bacteria and tuberculosis. Lancet Infect Dis.

[7] Bert F, Larroque B, Paugam-Burtz C (2012). Pretransplant fecal carriage of extended-spectrum β-lactamase-producing Enterobacteriaceae and infection after liver transplant, France. Emerg Infect Dis.

[8] Dubinsky-Pertzov B, Temkin E, Harbarth S (2019). Carriage of extended-spectrum beta-lactamase-producing Enterobacteriaceae and the risk of surgical site infection after colorectal surgery: a prospective cohort study. Clin Infect Dis.

[9] Willems RPJ, van Dijk K, Vehreschild M (2023). Incidence of infection with multidrug-resistant Gram-negative bacteria and vancomycin-resistant enterococci in carriers: a systematic review and meta-regression analysis. Lancet Infect Dis.

[10] Castanheira, M, Simner, PJ, Bradford, PA (2021) Extended-spectrum β-lactamases: an update on their characteristics, epidemiology and detection. JAC Antimicrob Resist 3 (3):dlab092. 10.1093/jacamr/dlab092 10.1093/jacamr/dlab092 PMC8284625 34286272

[11] Gomila A, Badia JM, Carratalà J (2017). Current outcomes and predictors of treatment failure in patients with surgical site infection after elective colorectal surgery. A multicentre prospective cohort study. J Infect.

[12] Ren Y, Ma G, Peng L, Ren Y, Zhang F (2015). Active screening of multi-drug resistant bacteria effectively prevent and control the potential infections. Cell Biochem Biophys.

[13] Budding AE, Grasman ME, Eck A (2014). Rectal swabs for analysis of the intestinal microbiota. PLoS ONE.

[14] Garcia ER, Vergara A, Aziz F (2022). Changes in the gut microbiota and risk of colonization by multidrug-resistant bacteria, infection, and death in critical care patients. Clin Microbiol Infect.

[15] McKinnell JA, Miller LG, Singh R (2016). Prevalence of and factors associated with multidrug resistant organism (MDRO) colonization in 3 nursing homes. Infect Control Hosp Epidemiol.

[16] Wong, SC, Chen, JH, Chau, PH, et al (2022) Gastrointestinal colonization of carbapenem-resistant acinetobacter baumannii: What is the implication for infection control? Antibiotics (Basel) 11(10). 10.3390/antibiotics11101297 10.3390/antibiotics11101297 PMC9598245 36289955

[17] Prado V, Hernández-Tejero M, Mücke MM (2022). Rectal colonization by resistant bacteria increases the risk of infection by the colonizing strain in critically ill patients with cirrhosis. J Hepatol.

[18] Xie J, Ma X, Huang Y (2014). Value of American Thoracic Society guidelines in predicting infection or colonization with multidrug-resistant organisms in critically ill patients. PLoS ONE.

[19] China Antimicrobial Surveillance Network. www.chinets.com. Accessed 25 Jan 2023

[20] Chen LF, Knelson LP, Gergen MF (2019). A prospective study of transmission of multidrug-resistant organisms (MDROs) between environmental sites and hospitalized patients-the TransFER study. Infect Control Hosp Epidemiol.

[21] Cohen B, Liu J, Cohen AR, Larson E (2018). Association between healthcare-associated infection and exposure to hospital roommates and previous bed occupants with the same organism. Infect Control Hosp Epidemiol.

[22] Rao, K, Patel, A, Sun, Y, et al (2021) Risk factors for Klebsiella infections among hospitalized patients with preexisting colonization. Msphere 6(3):e0013221. 10.1128/mSphere.00132-21 10.1128/mSphere.00132-21 PMC8265626 34160237

[23] Case definitions for public health surveillance. https://www.cdc.gov/mmwr/preview/mmwrhtml/00025629.htm. Accessed 2 May 2022

[24] CDC surgical site infection (SSI) event definition. http://www.cdc.gov/nhsn/pdfs/pscmanual/9pscssicurrent.pdf. Accessed 2 May 2022

[25] Jacobs D (2014). Clinical practice. Hemorrhoids N Engl J Med.

[26] Magiorakos AP, Srinivasan A, Carey RB (2012). Multidrug-resistant, extensively drug-resistant and pandrug-resistant bacteria: an international expert proposal for interim standard definitions for acquired resistance. Clin Microbiol Infect.

[27] CLSI Performance standards for antimicrobial susceptibility testing. 32nd ed. In stitute, Wayne, PA, USA: Clinical and Laboratory Standards Institution; 2022. https://www.standards-global.com/wp-content/uploads/pdfs/preview/2247002. Accessed 10 May 2022

[28] The European Committee on Antimicrobial Susceptibility Testing. Breakpoint tables for interpretation of MICs and zone diameters. Version 12.0, 2022. http://www.eucast.org. Accessed 12 May 2022

[29] Godonou AM, Lack F, Gbeasor-Komlanvi FA (2022). High faecal carriage of extended-spectrum beta-lactamase producing Enterobacteriaceae (ESBL-PE) among hospitalized patients at Sylvanus Olympio Teaching Hospital, Lomé, Togo in 2019. Afr J Clin Exp Microbiol.

[30] Mills EG, Martin MJ, Luo TL (2022). A one-year genomic investigation of Escherichia coli epidemiology and nosocomial spread at a large US healthcare network. Genome Med.

[31] Scott, NA, Andrusaite, A, Andersen, P, et al (2018) Antibiotics induce sustained dysregulation of intestinal T cell immunity by perturbing macrophage homeostasis. Sci Transl Med 10(464). 10.1126/scitranslmed.aao4755 10.1126/scitranslmed.aao4755 PMC6548564 30355800

[32] Lv Q, Xu D, Zhang X (2020). Association of hyperuricemia with immune disorders and intestinal barrier dysfunction. Front Physiol.

[33] Kalakouti E, Simillis C, Pellino G (2017). Characteristics of surgical site infection following colorectal surgery in a tertiary center: extended-spectrum β-lactamase-producing bacteria culprits in disease. Wounds.

[34] Molloy RG, Kingsmore D (2000). Life threatening pelvic sepsis after stapled haemorrhoidectomy. Lancet.

[35] Ripetti V, Caricato M, Arullani A (2002). Rectal perforation, retropneumoperitoneum, and pneumomediastinum after stapling procedure for prolapsed hemorrhoids: report of a case and subsequent considerations. Dis Colon Rectum.

[36] Di Domenico, EG, Farulla, I, Prignano, G, et al (2017) Biofilm is a major virulence determinant in bacterial colonization of chronic skin ulcers independently from the multidrug resistant phenotype. Int J Mol Sci 18(5). 10.3390/ijms18051077 10.3390/ijms18051077 PMC5454986 28513576

[37] Karanika S, Karantanos T, Arvanitis M, Grigoras C, Mylonakis E (2016). Fecal colonization with extended-spectrum beta-lactamase-producing Enterobacteriaceae and risk factors among healthy individuals: a systematic review and metaanalysis. Clin Infect Dis.

[38] Harris AD, McGregor JC, Johnson JA (2007). Risk factors for colonization with extended-spectrum beta-lactamase-producing bacteria and intensive care unit admission. Emerg Infect Dis.

[39] Bredin S, Charpentier J, Mira JP, Gastli N, Pène F, Llitjos JF (2022). Impact of colonization with multidrug-resistant bacteria on the risk of ventilator-associated pneumonia in septic shock. J Crit Care.

[40] Khan KI, Akmal M, Waqas A, Mahmood S (2014). Role of prophylactic antibiotics in Milligan Morgan hemorrhoidectomy - a randomized control trial. Int J Surg.

[41] Righi E, Mutters NT, Guirao X (2023). ESCMID/EUCIC clinical practice guidelines on perioperative antibiotic prophylaxis in patients colonized by multidrug-resistant Gram-negative bacteria before surgery. Clin Microbiol Infect.

[42] Phee L, Paget S, Jacques J, Bharathan B, El-Mugamar H, Sivaramakrishnan A (2021). Carbapenemase-producing organism (CPO) colonisation at a district general hospital: universal screening may help reduce transmission. Infect Prev Pract.

[43] Foschi C, Gaibani P, Lombardo D, Re MC, Ambretti S (2020). Rectal screening for carbapenemase-producing Enterobacteriaceae: a proposed workflow. J Glob Antimicrob Resist.

[44] Turner G, O'Grady M, Hudson D, Morgan X, Frizelle F, Purcell R (2022). Rectal swabs are a reliable method of assessing the colonic microbiome. Int J Med Microbiol.

[45] Blumetti J, Luu M, Sarosi G (2007). Surgical site infections after colorectal surgery: do risk factors vary depending on the type of infection considered?. Surgery.

[46] Wang J, Liu F, Tartari E (2018). The prevalence of healthcare-associated infections in Mainland China: a systematic review and meta-analysis. Infect Control Hosp Epidemiol.

[47] Hou TY, Gan HQ, Zhou JF (2020). Incidence of and risk factors for surgical site infection after colorectal surgery: a multiple-center prospective study of 3,663 consecutive patients in China. Int J Infect Dis.

[48] McCrink KA, DeRonde KJ, Jimenez A (2023). Impact of a real-time diagnostic and antimicrobial stewardship workflow on time to appropriate therapy for infections caused by multidrug-resistant Gram-negative organisms. Int J Antimicrob Agents.

